# A Competition, Benchmark, Code, and Data for Using Artificial Intelligence to Detect Lesions in Digital Breast Tomosynthesis

**DOI:** 10.1001/jamanetworkopen.2023.0524

**Published:** 2023-02-23

**Authors:** Nicholas Konz, Mateusz Buda, Hanxue Gu, Ashirbani Saha, Jichen Yang, Jakub Chłędowski, Jungkyu Park, Jan Witowski, Krzysztof J. Geras, Yoel Shoshan, Flora Gilboa-Solomon, Daniel Khapun, Vadim Ratner, Ella Barkan, Michal Ozery-Flato, Robert Martí, Akinyinka Omigbodun, Chrysostomos Marasinou, Noor Nakhaei, William Hsu, Pranjal Sahu, Md Belayat Hossain, Juhun Lee, Carlos Santos, Artur Przelaskowski, Jayashree Kalpathy-Cramer, Benjamin Bearce, Kenny Cha, Keyvan Farahani, Nicholas Petrick, Lubomir Hadjiiski, Karen Drukker, Samuel G. Armato, Maciej A. Mazurowski

**Affiliations:** 1 Department of Electrical and Computer Engineering, Duke University, Durham, North Carolina; 2 Department of Radiology, Duke University Medical Center, Durham, North Carolina; 3 Faculty of Mathematics and Information Science, Warsaw University of Technology, Warsaw, Poland; 4 Department of Oncology, McMaster University, Hamilton, Ontario, Canada; 5 Jagiellonian University, Kraków, Poland; 6 Department of Radiology, NYU Grossman School of Medicine, New York, New York; 7 Medical Image Analytics, IBM Research, Haifa, Israel; 8 Institute of Computer Vision and Robotics, University of Girona, Girona, Spain; 9 Medical and Imaging Informatics Group, Department of Radiological Sciences, David Geffen School of Medicine, University of California Los Angeles; 10 Department of Radiological Sciences, David Geffen School of Medicine, University of California Los Angeles; 11 Department of Bioengineering, University of California Los Angeles Samueli School of Engineering; 12 Department of Computer Science, Stony Brook University, Stony Brook, New York; 13 Department of Radiology, University of Pittsburgh, Pittsburgh, Pennsylvania; 14 Department of Radiology, Athinoula A. Martinos Center for Biomedical Imaging, Massachusetts General Hospital, Charlestown; 15 US Food and Drug Administration, Silver Spring, Maryland; 16 Center for Biomedical Informatics and Information Technology, National Cancer Institute, Bethesda, Maryland; 17 Department of Radiology, University of Michigan, Ann Arbor; 18 Department of Radiology, University of Chicago, Chicago, Illinois; 19 Department of Computer Science, Duke University, Durham, North Carolina; 20 Department of Biostatistics and Bioinformatics, Duke University Medical Center, Durham, North Carolina

## Abstract

**Importance:**

An accurate and robust artificial intelligence (AI) algorithm for detecting cancer in digital breast tomosynthesis (DBT) could significantly improve detection accuracy and reduce health care costs worldwide.

**Objectives:**

To make training and evaluation data for the development of AI algorithms for DBT analysis available, to develop well-defined benchmarks, and to create publicly available code for existing methods.

**Design, Setting, and Participants:**

This diagnostic study is based on a multi-institutional international grand challenge in which research teams developed algorithms to detect lesions in DBT. A data set of 22 032 reconstructed DBT volumes was made available to research teams. Phase 1, in which teams were provided 700 scans from the training set, 120 from the validation set, and 180 from the test set, took place from December 2020 to January 2021, and phase 2, in which teams were given the full data set, took place from May to July 2021.

**Main Outcomes and Measures:**

The overall performance was evaluated by mean sensitivity for biopsied lesions using only DBT volumes with biopsied lesions; ties were broken by including all DBT volumes.

**Results:**

A total of 8 teams participated in the challenge. The team with the highest mean sensitivity for biopsied lesions was the NYU B-Team, with 0.957 (95% CI, 0.924-0.984), and the second-place team, ZeDuS, had a mean sensitivity of 0.926 (95% CI, 0.881-0.964). When the results were aggregated, the mean sensitivity for all submitted algorithms was 0.879; for only those who participated in phase 2, it was 0.926.

**Conclusions and Relevance:**

In this diagnostic study, an international competition produced algorithms with high sensitivity for using AI to detect lesions on DBT images. A standardized performance benchmark for the detection task using publicly available clinical imaging data was released, with detailed descriptions and analyses of submitted algorithms accompanied by a public release of their predictions and code for selected methods. These resources will serve as a foundation for future research on computer-assisted diagnosis methods for DBT, significantly lowering the barrier of entry for new researchers.

## Key Points

**Question:**

Can a grand challenge be used to facilitate the advancement of automated digital breast tomosynthesis (DBT) cancer detection technology?

**Findings:**

This diagnostic study, in which 8 challenge teams developed algorithms to detect lesions on 22 032 DBT volumes, resulted in tumor detection performances as high as a mean biopsied lesion sensitivity of 0.957, which arose from the development of several novel approaches.

**Meaning:**

The variety of approaches that participants used in this study, alongside their released code and our released tumor detection benchmarking platform, present a starting point for future research in this area.

## Teaser

This diagnostic study describes a grand challenge for using artificial intelligence (AI) to detect cancer in digital breast tomosynthesis images.

## Introduction

Breast cancer is the leading cause of cancer death for women worldwide,^1^ and detection is a challenging process that requires the involvement of experienced radiologists. Digital breast tomosynthesis (DBT) creates high-resolution quasi–3-dimension (3D) scans consisting of multiple adjacent reconstruction slices, which reduces the effect of overlapping tissues seen in 2D mammography. This improves cancer detection rates but at the cost of increased reading time.^2^ An AI-based DBT cancer detection tool with radiologist-level performance could significantly reduce cancer screening costs and time and improve detection performance, which would be particularly helpful at sites that do not have access to fellowship-trained radiologists.

The most common AI method for image analysis is deep learning, which involves the training of nonlinear hierarchical models with many parameters (known as neural networks) to perform difficult tasks, such as image classification,^3^ object detection,^4^ and semantic segmentation,^5,6^ enabled by large data sets and specialized computing power.^7^ Deep learning detection algorithms have even surpassed radiologist performance^8,9^ due to their ability to learn far more complex features than earlier computer-assisted diagnosis systems, which had limited clinical applicability.^10,11^ In fact, algorithms with high sensitivity may even detect cancers missed by radiologists, serving as a second independent reader.^12^ However, developing deep learning algorithms for medical image analysis faces significant challenges, including a lack of sufficient, well-organized, and labeled training data; a lack of benchmark and test data as well as clearly defined rules for comparing algorithms, especially important because systems with significant false-positive rates can reduce radiologist sensitivity^12^; and limited access to previously developed algorithms for comparison. Moreover, DBT lesion detection introduces further difficulties for deep learning, including high scan resolution, high anatomical variability of both normal and abnormal breast tissue, and a very high class-imbalance of normal to cancerous cases for screening DBT.

In this article, we provide a practical foundation for the future open development and evaluation of algorithms for DBT lesion detection by providing a collection of analyses and resources for researchers, based on a new publicly available data set. Namely, we created a well-defined benchmark for evaluating future DBT lesion detection algorithms^13^; a description of several state-of-the-art algorithms for the task; a public release and comparative analysis of the predictions made by these algorithms, allowing for comparison with future approaches; and code for several of the algorithms, where possible. To generate these resources, we hosted a grand challenge, DBTex, for the automated detection of lesions in screening DBT scans. DBTex was divided into 2 phases, from December 14, 2020, to January 25, 2021, and May 24 to July 26, 2021, respectively. Challenges such as BraTS,^14^ ImageNet,^15^ other Kaggle competitions,^16^ and others have long been used to move the field forward by motivating intense and competitive research.

Several recent works have used deep learning to either classify DBT scans for the presence of lesions^17,18,19,20,21,22,23,24,25,26,27,28,29^ or localize lesion(s) within DBT scans. Localization tasks include determining the exact shape of these lesions, known as segmentation,^12,30,31^ or drawing bounding boxes around them, known as detection.^32,33,34,35,36,37,38,39,40^ Our challenge task was the detection of masses and architectural distortions in DBT scans.

Challenge teams developed and trained their detection methods on a large data set of healthy participants, with limited scans containing lesions, from a recently released large, public radiologist-labeled data set of DBT volumes from 5060 patients. After the training phase, participants were provided with a smaller validation data set to fine-tune their methods. At the end of the challenge, teams applied their methods to a previously unseen test set of scans with normal and cancerous tissue, which was used to obtain final rankings. While pathology and lesion locations of the training set were shared with participants as a reference standard, they were made unavailable for the validation and test sets.

## Methods

This study was approved by the Duke University Health System institutional review board with a waiver of informed consent due to its retrospective nature. The Duke University Breast Cancer Screening DBT (BCS-DBT) data set, which was provided by the challenge organizers, was publicly available data. Three teams used additional data: the NYU B-Team used an internal data set approved by the NYU Langone Health institutional review board, ZeDuS used an internal institutional review board–approved data set, and VICOROB used the OPTIMAM data set (OMI-DB), whose ethical approval is publicly available.^41^ This study followed the Transparent Reporting of a Multivariable Prediction Model for Individual Prognosis or Diagnosis (TRIPOD) reporting guideline.

### Data Set

DBTex was built on Duke University’s BCS-DBT data set,^32^ which was designed to be the first large, curated and labeled, and publicly available DBT data set, hosted on the Cancer Imaging Archive.^42,43^ It includes 22 032 reconstructed DBT volumes (a stack of spatially adjacent 2D scan slices) from scans of 5060 participants, with annotations for biopsied lesions provided by 2 trained radiologists. A given DBT scan has separate volumes corresponding to at least 1 and as many as 4 of the anatomical views of the breasts: left craniocaudal (LCC), right craniocaudal (RCC), left mediolateral oblique (LMLO), and right mediolateral oblique (RMLO).

Each of the radiologists who completed the annotations had at least 18 years of experience with breast imaging. Scans were classified as normal, actionable (further imaging requested), benign (lesion found, negative biopsy), or cancerous (lesion found, positive biopsy). Additionally, for benign and cancerous cases, radiologists provided annotations in the form of a tight bounding box around each lesion. If a lesion annotation were present in a volume, the annotation was assigned to the central slice of the volume. (There are approximately 70 slices for each scan volume.) Annotations for microcalcifications were not included. For the challenge, the data set was stratified by participant into training, validation, and test sets, as outlined in Table 1. Lesion boxes and volume class labels were only provided to challenge teams for the training set. In phase 1 of the challenge, teams were provided with 700 scans from the training set, 120 from the validation set, and 180 from the test set, while the second phase used the entire data set. All lesion cases were included in both phases. We provide further logistical details for the challenge in eAppendix 2 in Supplement 1.

**Table 1.** Statistics of the Data Sets Used for the Challenge

Characteristics	No. (%)		
	Training set	Validation set	Test set
Participants			
Total	4362 (100)	280 (100	418 (100)
Normal	4109 (94.2)	200 (71.4)	300 (71.8)
Actionable	178 (4.1)	40 (14.3)	60 (14.4)
Benign	62 (1.4)	20 (7.1)	30 (7.2)
Cancer	39 (0.9)	20 (7.1)	30 (7.2)
Total DBT volumes, No.	19 148	1163	1721
Bounding boxes for biopsied lesions, No.	224	75	136

Abbreviation: DBT, digital breast tomosynthesis.

### Statistical Analysis

Teams were tasked with developing algorithms that take a DBT volume as input and detect any biopsy-proven (cancerous or benign) lesions found within by generating proposed bounding boxes that enclose the lesion(s). To evaluate this task on the validation and test sets of scans with class labels and lesion bounding boxes unknown to participants, teams were asked to provide bounding box locations (horizontal and vertical pixel coordinates and slice index) and sizes, accompanied by prediction scores indicating a level of certainty for each box for any lesions detected by their models. These scores could be on any scale, but the scale had to be unified across the evaluation of the data set and were used by challenge organizers (along with the bounding boxes submitted by participants and reference standard) to evaluate the overall detection performance of an algorithm.

The overall performance evaluation for an algorithm was based on free-response receiver operating characteristic (FROC) curves, which examine the sensitivity of each model with respect to the number of false-positive (FP) predictions created by the model for each view in the test set. Details of how a prediction was deemed a true positive appear in eAppendix 2 in Supplement 1. The primary performance metric was calculated only on DBT volumes with biopsied lesions (benign or malignant) and was the mean sensitivity (ie, the true-positive rate) over 1, 2, 3, and 4 FPs per volume. We average over the different FP counts to comprehensively reflect the overall performance curve across different sensitivities and specificities. This metric is similar to the competition performance metric for assessing lung nodule detection.^44^

The secondary performance metric used to break ties, if any, was the sensitivity at 2 FPs per DBT volume calculated using all DBT volumes (eTable in Supplement 1). To win the challenge, a team’s performance did not need to demonstrate a statistically significant improvement over that of the runner-up. The number of views in the test set with biopsied lesions was the same in both challenges, meaning that the primary performance metric is identical. All evaluation code is publicly available.^45^ Finally, we created a webpage for future evaluations of the DBTex performance metric (on both the validation and test sets).^13^ This will allow algorithms developed in the future to have a standardized tool for model selection (by the validation set) and performance metric (on the test set).

## Results

### Grand Challenge Results

In Table 2, we present the ranked roster of participating teams: their affiliations, method names, final scores for all challenge phases that they participated in, and a reference to their code when possible.^45,46,47,48,49,50,51,52,53,54,55,56^ We also provide the performance results of 2 simple baseline models on the task, both with code: the model that accompanied the BCS-DBT data set release^32^ and a basic faster region-based convolutional neural network (R-CNN) model (eAppendix 2 in Supplement 1). We provide detailed summaries of all algorithms in eAppendix 2 in Supplement 1, with a link to all prediction results. The teams that participated in both challenge phases had the same final ranking order, so we display the results of both phases in the same table. eAppendix 1 in Supplement 1 presents secondary metric results.

**Table 2.** Challenge Results^a^

Ranking	Team name	Affiliations	Methods	Training set	Mean sensitivity for biopsied lesions (95% CI)	Phase where team achieved best performance	Code available
1	NYU B-Team	New York University—Langone Health	Phase 1: EfficientDet, Max-Slice-Selection, and Augmentation and Ensembled Perturbations; phase 2: phase 1 methods with cancer cell prediction head and multilocation crop	Phases 1 and 2: DBTex1 and internal data set	0.957 (0.924-0.984)	2	No
2	ZeDuS	IBM Research—Haifa	Phase 1: RetinaNet ensemble with heatmap NMS; phase 2: phase 1 methods with SWIN^46^ and NFNet^47^	Phases 1 and 2: DBTex1 with internal data set	0.926 (0.881-0.964)	2	Yes, both phases^48^
3	VICOROB	VICOROB—University of Girona	Phase 1: Fast R-CNN, ensembled; phase 2: phase 1 methods with FP reduction (no ensemble)	Phases 1 and 2: DBTex1 with OPTIMAM/OMI-DB	0.886 (0.836-0.930)	2	Yes, both phases^49,50^
4	Prarit	Queen Mary University of London—CRST and School of Physics and Astronomy	Unknown	Unknown	0.822 (0.754-0.884)	1	No
5	UCLA-MII	UCLA Medical & Imaging Informatics	Phase 1: Faster R-CNN, FPN,^51^ IoSIB, and Blob Detector	Phase 1: DBTex1	0.814 (0.751-0.875)	1	Yes, phase 1^52^
6	Pranjalsahu	Stony Brook—Department of Computer Science	Phase 1: Faster R-CNN with Confidence Peak Finder	Phase 1: DBTex1	0.790 (0.717-0.854)	1	Yes; phase 1^53^
7	Team-PittRad	University of Pittsburgh—Department of Radiology	Phase 1: YOLOv5^54^ and Cross Stage Partial Networks	Phase 1: DBTex1	0.786 (0.720-0.852)	1	Yes, phase 1^55^
8	Coolwulf	Unknown	Unknown	Unknown	0.390 (0.301-0.475)	1	No
NA	Baseline model^b^	NA	Faster R-CNN	DBTex1	0.379 (0.304-0.456)	NA	Yes^56^
NA	Data set baseline model^b^	NA	DenseNet^32^	DBTex1	0.444 (0.366-0.523)	NA	Yes^45^

Abbreviations: FP, false positive; FPN, feature pyramind network; IoSIB, intersection over the smaller intersecting box; NA, not applicable; NFNet, Normalizer-Free-ResNets; NMS, nonmaximal suppression; R-CNN, region-based convolutional neural network; SWIN, shifted window transformer.

^a^ 95% confidence intervals (CI) were computed using bootstrapping, with 5000 bootstraps.

^b^ Not submitted for challenge.

### Analysis of Grand Challenge Results

Beyond the individual performances of each participant algorithm, we analyzed the collective results of the challenge to obtain a holistic measure for the capability of state-of-the-art DBT lesion detection algorithms. First, we examined how lesion detection difficulty varied between different cases. Next, we analyzed the success of the submitted algorithms by aggregating their predictions.

#### Lesion Detection Difficulty Ranking

**Figure 1.**
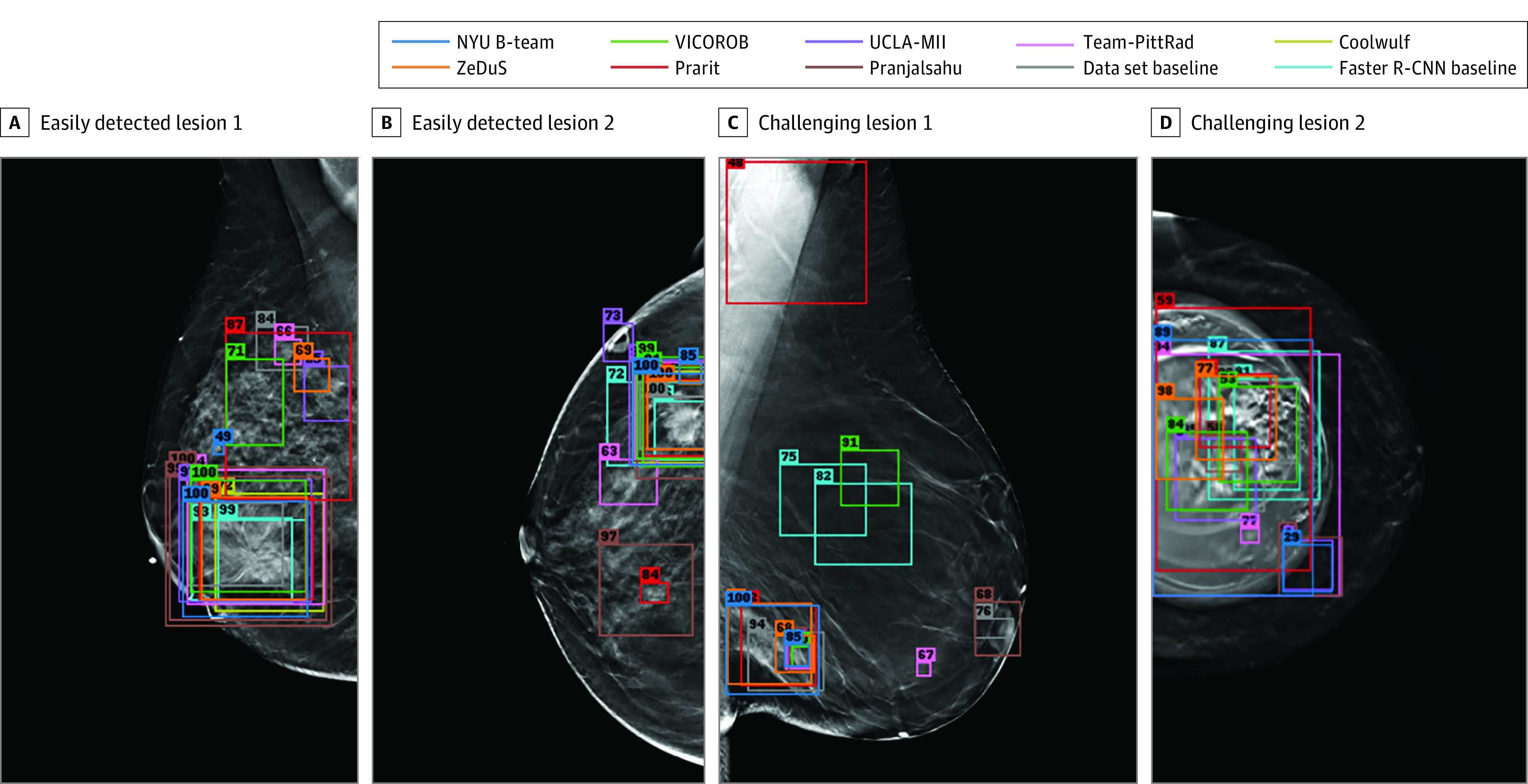
The Least and Most Difficult Lesions to Detect A and B, Examples of digital breast tomosynthesis volumes containing annotated lesions that were the easiest to detect. On average, all 10 algorithms detected lesions in A and with 0.13 and 0.16 false positives, respectively. C and D, Examples of digital breast tomosynthesis volumes containing annotated lesions that were the most difficult to detect. The lesion in panel C was not detected by any algorithm, and the lesion in panel D was detected by only 2 of 10 algorithms with 1.34 false positives on average (due to the presence of a breast implant). Detection bounding boxes indicate submitted algorithm predictions. The number in the upper-left corner of each box indicates the percentile of the corresponding algorithm’s score with respect to the distribution of all algorithm scores for the volume. At most, 2 boxes per algorithm are shown, and the colors of each algorithm’s boxes correspond to the free-response receiver operating characteristic curves shown in Figure 2.

We considered 2 extremes: the easiest lesions to detect and the most difficult. Figure 1A and 1B show the 2 lesions that were easiest to detect according to our difficulty measures (eAppendix 2 in Supplement 1), alongside the algorithms’ corresponding predictions. We see that most submitted algorithms made similar predictions for these lesions. Figure 1C and D show the 2 most challenging lesions, which resulted in disagreeing predictions. Overall, we found that within 4 FPs per DBT volume all algorithms detected 16 of 136 lesion annotations (12%), and there was only 1 lesion (<1%) that was not detected by any algorithm.

Next, we analyzed the association of lesion detection difficulty with (1) the classification of the lesion being cancerous or benign and (2) lesion type (mass or architectural distortion). We compare these lesion characteristics with our measures of lesion difficulty in Table 3. We also measured the correlation between the number of algorithms that detected a lesion and the lesion size (bounding box diagonal length) to be 0.28, using Spearman rank correlation coefficient.

**Table 3.** Comparison of Lesion Detection Difficulty Metrics and Lesion Characteristics for the Test Set

Metric	Mean (SD)			
	Lesion diagnosis		Lesion type	
	Benign	Cancer	Mass	Architectural distortion
Total count, No.	70	66	121	15
No. of algorithms that detected lesion within 4 FPs per volume	7.47 (1.73)	7.82 (1.76)	7.63 (1.78)	7.73 (1.44)
FPs corresponding to correct prediction considering teams that detected it within 4 FPs per volume	0.77 (0.49)	0.55 (0.43)	0.64 (0.47)	0.91 (0.47)

Abbreviation: FP, false positive.

#### Combining Predictions

To aggregate lesion detection results from all methods (not including the baseline models), ie, to merge lesion bounding box predictions that were made by different algorithms, we normalized the detection confidence scores assigned by each algorithm to its predicted bounding boxes, across all algorithms, by transforming all scores for each algorithm to a percentile range. Next, we merged lesion box predictions across the width, height, and slice dimensions using the weighted boxes fusion algorithm^57^ (eAppendix 2 in Supplement 1).

**Figure 2.**
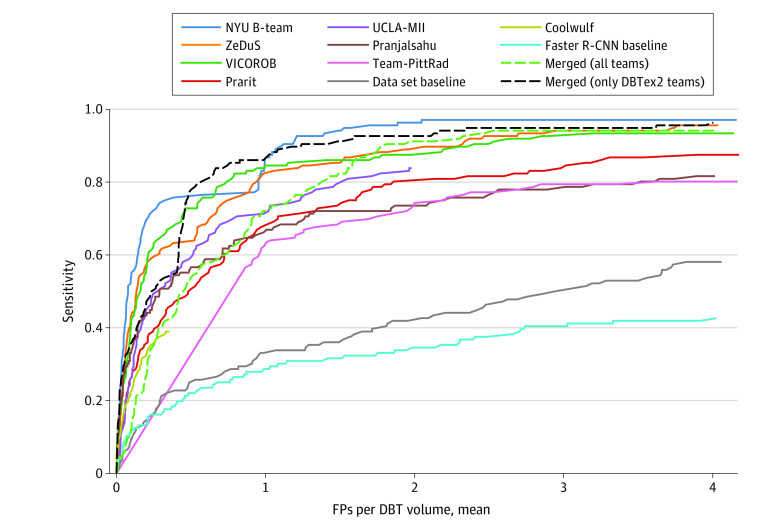
Free-Response Receiver Operating Characteristic Detection Curves for All Methods Includes all participants, baseline models, and merged predictions from all algorithms and only the top 3 models from phase 2.

After this merging procedure, we obtained a set of merged predicted lesion bounding boxes with accompanying prediction scores aggregated from each model. We computed merged results for both (1) all submitted algorithms and (2) only phase 2 submissions. For the 3 teams that participated in both phases, we use the results from phase 2, as they were superior to phase 1 submissions in all cases. The mean sensitivity at 1, 2, 3, and 4 FPs per volume was 87.9% (95% CI, 83.6%-91.8%)for the former group and 92.6% (95% CI, 88.8%-95.9%) for the latter. On the same metric, for phase 2, NYU B-Team achieved 94.3% (95% CI, 90.7%-97.2%); ZeDuS, 90.4% (95% CI, 86.1%-94.3%); and VICOROB, 89.6% (95% CI, 85.0%-93.7%). We show these results as FROC curves in Figure 2, including the Faster R-CNN baseline and the baseline model from Buda et al.^32^

## Discussion

The purpose of this challenge was to facilitate the development of state-of-the-art machine learning algorithms for the challenging task of DBT lesion detection. Our goal was to address the current lack of public, standardized resources for research in this field, as prior works have often relied on private data sets, detection models, or both. We approached this by (1) establishing a standardized, publicly accessible testing benchmark, evaluation pipeline, and training data set for this task; (2) hosting a grand challenge to encourage the concentrated development of algorithms; and (3) encouraging the release of publicly available algorithm code.

By merging the results from all submissions, we found that most submitted algorithms achieved strong performance on this task. All submitted algorithms that we analyzed (eAppendix 2 in Supplement 1) relied on some object detection neural network. The key properties that led some submissions to perform better than others were (1) the specific model used, (2) any novel refinements that teams made to their methods, and (3) the training data used. Leading teams used more recent detection architectures, such as EfficientDet^58^ (NYU B-Team), highly optimized for modern object detection tasks, or RetinaNet^37^ (ZeDuS), which is especially well-suited for small objects (eg, small lesions) and class-imbalanced data sets like the BCS-DBT training set. The top 3 teams also used model ensembling, the aggregation of multiple models’ predictions to improve overall robustness. This method is well-suited for improving the generalizability of models,^59^ which is especially applicable to this task because breast tissue and breast lesions have high morphological variability. Finally, winning teams also used additional internal training data that provided more lesion examples to learn from. This is important because the detection model itself can only carry performance so far; the training of deep models is data driven, so the variety and quantity of lesion examples to learn from will significantly affect an algorithm’s ability to generalize to new data.

The importance of training data, model choice, and novel methods is especially apparent when submitted results are compared with the 2 baseline models (Table 2), which were trained only on the provided BCS-DBT training set and had poorer performance than most submissions. However, some submissions (eg, UCLA-MII, pranjalsahu, Team-PittRad) that also only used the provided data performed almost as well as algorithms that used additional data, while vastly outperforming the baseline models (Table 2). As such, the usage of supplementary data appears not to be the only necessary factor for achieving good performance, but also the development of specialized techniques for the unique characteristics of the data set or domain, which the baseline models did not have. This shows that DBT tumor detection is a challenging problem for typical detection models, but large performance improvements are possible if the model development is targeted for this modality. Finally, an additional tactic that some teams used (eg, VICOROB and UCLA-MII) was pretraining their detection models on common universal natural image data sets, such as COCO,^60^ before training on the target domain of DBT data, giving the models a starting point for visual feature recognition.

### Limitations

This study has limitations. All teams relied on supervised training of their detection algorithms, ie, directly recognizing visual features that discriminate between healthy and cancerous examples. One potential obstacle for this approach is the presence of anomalous objects that may mislead detection. The case shown in Figure 1D is an example of this, where an implant distracted most of the models from the mass present in the image. This behavior is due to the data-driven nature of training deep learning models; if an object appears in a test image that is rare within the training data, this may interfere with model predictions. Despite the strong overall results of the algorithms, the presence of these rare cases cannot be ignored in the clinical setting; this could be mitigated by the use of anomaly detection methods.^61,62^

Another limitation is that each of the top 3 finalists used additional training data including lesions outside of the provided data set, so no conclusion can be drawn about which method would be superior given the same training data set. However, submitted algorithms were still directly compared in their effectiveness at detecting lesions in the test set. An additional limitation of our study is that the benchmark was computed only on true-positive cases. This was because the data set has missing views but only for biopsied cases, which could be (intentionally or not) taken advantage of by participants by prioritizing predictions for these missing-view cases or determining which cases are biopsied by the presence of missing views. However, by comparing the competition results (Table 2) with the FROC curves in Figure 2, the latter of which were computed on all cases, we found there to be no notable difference between (1) the metric computed on all cases and (2) only true-positive cases, so the effect of this factor is minimal.

The scope of the test set was somewhat limited because it included only 136 lesions (due to the natural screening rarity of breast cancer) and because microcalcification annotations are not included in the data set.^32^ The ability of our benchmark to measure clinical detection performance across a range of institutions is also limited because the data originated only from the Duke University Health System. However, the leading algorithms’ success of using supplementary training data indicates that DBT scans created at different sites still possesses common features to learn from and implies that the algorithms were able to generalize across multiple data domains.

## Conclusions

In this diagnostic study of AI for DBT, submitted algorithms for the DBTex challenge gave promising breast lesion detection performance over a range of difficult cases, improving over existing baseline models by a wide margin. To accompany these results, we presented a benchmark evaluation platform for assessing detection algorithms, a large public data set, and code for certain submitted algorithms. The success of this challenge marks a large improvement in DBT tumor detection methods, and the public resources we provide lay the groundwork for the development of clinically relevant computer-assisted diagnosis systems.

## Notes

Supplement 1. **eAppendix 1.** Additional Challenge Results **eTable.** Secondary Metric Challenge Results **eAppendix 2.** Additional Methods Details

Supplement 2.
Data Sharing Statement
