# Supplementary material for: A Competition, Benchmark, Code, and Data for Using Artificial Intelligence to Detect Lesions in Digital Breast Tomosynthesis
Source: JAMA Netw Open. 2023 Feb 23;6(2):e230524. doi: 10.1001/jamanetworkopen.2023.0524 (PMC9951043; doi:10.1001/jamanetworkopen.2023.0524)
Supplement: Supplement 1. — eAppendix 1. Additional Challenge Results eTable. Secondary Metric Challenge Results eAppendix 2. Additional Methods Details [file jamanetwopen-e230524-s001.pdf]

Supplemental Online Content

Konz N, Buda M, Gu H, et al. A competition, benchmark, code, and data for using artificial intelligence to detect lesions in digital breast tomosynthesis. *JAMA Netw Open.* 2023;6(2):e230524. doi:10.1001/jamanetworkopen.2023.0524

eAppendix 1. Additional Challenge Results

eTable. Secondary Metric Challenge Results

eAppendix 2. Additional Methods Details

This supplemental material has been provided by the authors to give readers additional information about their work.

© 2023 Konz N et al. *JAMA Network Open.*

eAppendix 1. Additional Challenge Results

See eTable 1 for the secondary performance metric results (sensitivity at 2 FPs per DBT volume calculated using *all* DBT volumes) for each teams' submitted algorithm.

© 2023 Konz N et al. *JAMA Network Open.*

eTable. Secondary Metric Challenge Results (including both phases and baseline algorithms), ordered by primary metric performance (Table 2). 95% confidence intervals (CI) were computed using bootstrapping, with 5000 bootstraps. Refer to Table 2 for primary performance metric results.

Ranking	Team Name	Affiliation(s)	Secondary performance metric: sensitivity at 2 FPs/image for all images (see text) with 95% CI
1	NYU B-Team	New York University – Langone Health	**Phase 1:** 0.912 (0.862; 0.954)<br>**Phase 2:** 0.956 (0.921; 0.987)
2	ZeDuS	IBM Research – Haifa	**Phase 1:** 0.900 (0.850; 0.947)<br>**Phase 2:** 0.893 (0.839; 0.943)
3	VICOROB	VICOROB – University of Girona	**Phase 1:** 0.875 (0.818; 0.930)<br>**Phase 2:** 0.868 (0.812; 0.920)
4	prarit	Queen Mary University of London – CRST and School of Physics and Astronomy	**Phase 1:** 0.805 (0.730; 0.874)
5	UCLA-MII	UCLA Medical & Imaging Informatics	**Phase 1:** 0.838 (0.771; 0.896)
6	pranjalsahu	Stony Brook – Department of Computer Science	**Phase 1:** 0.735 (0.655; 0.813)
7	Team-PittRad	University of Pittsburgh – Department of Radiology	**Phase 1:** 0.743 (0.657; 0.813)
8	coolwulf	*Unknown*	**Phase 1:** 0.390 (0.301; 0.475)
N/A	Baseline model (not submitted for challenge)	N/A	**Phase 2:** 0.346 (0.264; 0.429)
N/A	Dataset baseline model (not submitted for challenge)	N/A	**Phase 2:** 0.423 (0.339; 0.512)

© 2023 Konz N et al. *JAMA Network Open.*

eAppendix 2. Additional Methods Details

B.1. Grand Challenge Set-Up

DBTex was organized by the Duke Center for Artificial Intelligence in Radiology (DAIR), the International Society for Optics and Photonics (SPIE), the American Association of Physicists in Medicine (AAPM), and the National Cancer Institute (NCI)—the latter three having a history of organizing a number of SPIE-AAPM-NCI Grand Challenges. We used The Medical Imaging Challenge Infrastructure (MedICI, https://www.medici-challenges.org/), which provides several useful features for the challenge. With MedICI, the duration of a challenge can be divided into *phases*; DBTex used a *training phase*, a *validation phase,* and a *testing phase*. The training phase began with the release of the training set with class labels and lesion bounding boxes and lasted about three weeks for both Phase 1 and Phase 2. This phase allowed participants to begin building and training their models. For Phase 2, the validation and test sets were also released at the beginning of this phase (only scans, no annotations), while in Phase 1, these sets were not released until the start of their corresponding phases. The websites for Phase 1 and Phase 2 can be found at https://www.aapm.org/GrandChallenge/DBTex/ and https://www.aapm.org/GrandChallenge/Phase 2/, respectively.

The training phase was followed by the validation phase, which gave teams some number of model evaluations on the validation set (50 for Phase 1, 100 for Phase 2), using the evaluation procedure described in Section 1.2. Teams could evaluate their models without seeing class labels or lesion boxes because the evaluation system was embedded within the challenge platform, to which the teams could submit their algorithms' predictions. This phase allowed teams to test their trained models on unseen data in order to refine or select their models and perform hyperparameter tuning. It lasted eleven days for Phase 1 and about a month for Phase 2. Finally, the test phase, which lasted ten days for both Phase 1 and Phase 2, is where the teams submitted results to be used for the ultimate ranking; the validation phase was used for model selection without relying on the test set. In this challenge, teams were only given three attempts to evaluate the performance of their model on the unseen test set to avoid teams gleaning information from the test data through extensive evaluation. The top three Phase 1 teams presented their results during the 2021 SPIE Medical Imaging Symposium special challenge session. The top two Phase 2 teams presented at the Grand Challenges Symposium session of the 2021 AAPM Annual Meeting.

© 2023 Konz N et al. *JAMA Network Open.*

B.2. Submitted Algorithms

In this section, each participating team describe their submitted algorithm: (1) their model/methodology, (2) the data used, and (3) any changes to their method made between the first and second phases of the challenge, if they participated in both. The validation set and test set prediction data of all algorithms, for Phase 1 and Phase 2, are provided at https://wiki.cancerimagingarchive.net/pages/viewpage.action?pageId=64685580 under "DBTex Lesion Detection Challenge Test Set Predictions". Refer to Table 2 for a summary of each algorithm, its results for the challenge, and links to code where applicable.

NYU B-Team (Rank 1)

Model

The NYU B-Team developed an object detection system based on the EfficientDet[44] architecture. We augmented the input images using affine transformations, horizontal flips, and copy-paste segmentations to have multiple bounding box annotations for each image during training. For lesion detection, we defined an additional head that was trained to predict whether a box contains cancer cells and provided an additional training signal to the model. We post-processed the model output using several different methods, including a modification of the technique used in Buda et al. 2021. One of such postprocessing methods was our novel *max-slice selection*, where we selected the slice with the maximum confidence score for each anchor. Our submission was an ensemble of several models trained with slightly perturbed training sets for increasing diversity within the ensemble.

Data

We collected a dataset of 100,000 internal DBT exams along with pixel-level lesion annotations of biopsied lesions. Pixel-level labels were acquired with ITK-SNAP software and later converted to box coordinates for training and evaluating object detection systems. We also utilized 200,000 internal Full-Field Digital Mammography (FFDM) exams and the BCS-DBT training set.

Additions for Phase 2

© 2023 Konz N et al. *JAMA Network Open.*

Hyperparameters. Compared to the first challenge, we changed the following hyperparameters:

- In the Focal loss (Lin et al., 2017b), we use an α value of 0.75-0.85 instead of 0.25.
- Also, in the Focal loss, instead of treating predictions with <0.4 IoU as negative and ≥0.5 IoU as positive, we treat predictions with <0.2 IoU as negative and ≥0.6-0.7 IoU as positive.

Model Addition. We defined an additional head that is trained to predict whether a box contains cancer cells and provided an additional training signal to the model.

Inference. We cropped a smaller portion of the DBT slices to make the training more efficient. In the first challenge phase, we cropped from the most informative region during inference. However, this made the risk of the algorithm missing lesions outside of the cropping window possible. In the second phase, we cropped multiple locations of the volume and aggregated the predictions to cover the entire breast during inference.

Additional Augmentations and Data. We added copy-paste augmentations[49] to the pool of augmentations. There was also an update to our additional dataset of DBT exams. After the update, compared to the DBT dataset we used in the first challenge, our DBT dataset contained 733 additional DBT exams (2,979 additional volumes). We also acquired additional segmentations for 1,397 volumes.

ZeDuS (Rank 2)

Model

We used an ensemble of 12 RetinaNet detection models[36], with varied hyper parameters such as number of layers and their width, and different augmentation methods such as intensity of random rotation to perform lesion localization. To combine detection predictions from different models we created a heatmap-based detectors ensembling method which augments NMS (non-maximum suppression). In our method, a heatmap was rendered using the predicted boxes, and pixel values accumulated the confidence scores of intersecting boxes from the different detectors. For further details on the detectors ensemble method see Shoshan et al., 2021 and the linked code in the second row of Table 2.

Data

In addition to the BCS-DBT dataset, we used an in-house dataset of 2D and 3D (DBT) mammograms of over 9,000 women from the USA and Israel. Over 1,000 of these exams had biopsy-proven cancer, and all biopsied cases contained localized contour annotations.

Additions for Phase 2

We added a transformer-based detector, SWIN[50], to our ensemble. We also implemented a slice classifier based on NFNet[51] which predicted a malignancy scalar per slice in a given DBT volume. To combine the slice classifier with the detector models, prediction boxes that were found on a slice were multiplied by the slice classifier confidence, to take into account both types of models.

VICOROB (Rank 3)

Model

VICOROB's detection algorithm was based on a Faster R-CNN architecture[4] ensembled using two different backbones: a ResNet-101[35] and a Res2Net[52]. The detection algorithm was applied at a 2D slice basis in a full DBT volume. The model was first fine-tuned on an internal mammographic image database and then fine-tuned again using the BCS-DBT training dataset. For inference, the model was applied at each volume slice (except the first and last four slices, as it was considered too close to the skin) to obtain the candidate bounding boxes. The final bounding boxes were obtained after post-processing the candidate boxes, according to a minimum score and overlap. The overlap between neighboring boxes (multiple detections) was minimized using an IoU (intersection over union) criteria. A simple ensemble was built combining the predictions of the two different Faster R-CNN architectures with ResNet101 and Res2Net backbones. The algorithm was implemented in PyTorch[53], using the Detectron2 framework[54] and the FastAI library[55].

Data

© 2023 Konz N et al. *JAMA Network Open.*

The Faster R-CNN model was initialized with the pre-trained weights of the COCO database[46]. Next, we fine-tuned the model with a subset of the OPTIMAM mammographic image database[56] using 3,614 malignant lesions (OMI-DB). Finally, the model was fine-tuned again with the BCS-DBT training set.

Additions for Phase 2

False Positive Reduction

Although the primary detection method was the same as the first phase (Faster R-CNN), instead of using a (small) ensemble as we did in Phase 1, we applied a false positive (FP) reduction step to the lesion bounding boxes candidates and used a larger dataset of cases including benign cases (Phase 1 only included malign cases). For the FP reduction step, an image classification algorithm was trained on false positive and true positive DBT detections (extracted from the training data in a cross-validation fashion). This step helped reduce the number of FPs of the detection process.

Additional Data

In total, 6,794 cases (all mammography, no DBT) with benign and malignant lesions were used for training. As in Phase 1, the classifier was fine-tuned with the BCS-DBT cases.

UCLA-MII (Rank 5, Phase 1 only)

Model

The algorithm for our submission had three main stages: a first stage to detect biopsied tissue in DBT slices with a convolutional neural network (CNN) detector, followed by a stage to reconstruct biopsied tissue volumes from detections in slices, and a final step to remove false-positive predictions with clinically significant skin markers using a detector with simple geometric rules.

2D (In-slice) Lesion Detection Ensembling

We utilized Faster R-CNN[4], a 2D detector with a Feature Pyramid Network[57] feature extractor, and a Resnet-50[35] backbone. The model was initialized with weights from pre-training with the COCO train2017 data

© 2023 Konz N et al. *JAMA Network Open.*

split[46]. The input to the model was three consecutive slices that were concatenated as a three-channel image. For training, slices around the lesion centers z (in the range z-3 to z+3) were used with random flipping, brightness changes, and gamma adjustment augmentations applied. To monitor the performance during training, a portion of the training set was held out (20% patient-wise). The training set was split into five parts, and five models were trained in a cross-validation scheme. The mean sensitivity on the held-out data was 0.780 ± 0.07 at two false positives per slice. During inference on the Phase 1 validation and testing data, all five models were applied to every slice, generating five sets of bounding box predictions for each slice. Detections from the three individually best of five models on the Phase 1 validation data were selected for the final ensemble[58], as the mean sensitivity was better than with all five models.

3D Lesion Candidate Generation

We then took the 2D in-slice bounding box predictions and combined them into 3D candidates based on: (1) the proximity of slices in a DBT scan (being within 50% of the total number of slices of each other), (2) 2D bounding box score threshold (at least 0.85 on a scale of 0 to 1), and (3) 2D bounding boxes with an intersection over the smaller intersecting box (IoSIB) greater than 0.75. The IoSIB gave better results than the standard intersection over union (IoU). 3D candidates with a depth of 1 (a single slice) were removed. The score assigned to a 3D candidate was the average of the top 10 scores of all 2D bounding boxes involved in its construction.

Blob Detection for False Positive Removal

To remove false positives (with clinical circular markers), we used the SimpleBlobDetector in the python OpenCV module, cv2[59], with the following parameter settings: filterByArea=True, minArea=1000, filterByConvexity=True, minConvexity=0.001, maxConvexity=0.4, filterByInertia=True, minInertiaRatio=0.01, maxIntertiaRatio=1, minThreshold=50 ([0,255] range), maxThreshold=150 ([0,255] range).

pranjalsahu (Rank 6, Phase 1 only)

Model

Our method used a Faster R-CNN[4] object detection model with ResNet-50[35] as a feature extraction backbone. Only the last three backbone layers were trained using the DBT dataset. Augmentation techniques such as

random horizontal and vertical flipping, rotation, and zoom were applied while training. Also, since ResNet-50 takes three-channel images, we took three consecutive slices from the volume as input. The training slices were extracted using the pre-processing function given with the challenge. Finally, the slices were divided by 60,000 for normalization, and clipped to a range of [0, 1].

We used the RMSProp optimizer[60] with a constant learning rate of 0.00001 to optimize the network parameters. We also used a weight decay of 0.001 to regularize and avoid overfitting. Initially, the network was trained with 175 samples, and the remaining 49 samples were used as validation samples. This training step provided the number of epochs needed to train the network. In the next step, the network was trained with all 224 samples for the same number of epochs to obtain the challenge validation set results.

The task was detecting the lesions within a volume; therefore, the results from the individual slices needed to be combined. For this purpose, we obtained one lesion prediction from Faster R-CNN having the highest confidence score from each slice. Next, starting from the first slice and until the last slice in the volume, we plotted the bounding box along with its corresponding score (probability). When the bounding box center for two consecutive slices was more than 10 pixels apart, we record a confidence of 0; otherwise, the score of the current slice was recorded. Next, we determined the lesion center coordinates by finding the peaks of this confidence score plot, using the find_peaks method of the scipy.signal Python library[61]. Finally, the bounding box of the obtained center slice was taken as the in-plane width and height for the lesion, and a fixed height of 10 pixels was taken in the slice dimension.

Team-PittRad (Rank 7, Phase 1 only)

Model

It is common to use large-scale external datasets to develop deep learning models. The number of positive cases in the Phase 1 training set was limited. To tackle this, we hypothesized that false-positive findings (FPs) from non-biopsied (actionable) lesions could help the algorithm as an alternative to using large in-house datasets. We first extracted 2D slices from DBT volumes of biopsy-proven (cancer and benign) and actionable cases as preprocessing. We then synthesized 2.5D images by merging the lesion center slice with neighboring slices to keep the lesion

continuity along the slice direction. We developed our breast lesion detection framework based on a single-stage object detector[38], YOLOv5[62] with Cross Stage Partial Networks[63] as a backbone. We constructed two baseline detection algorithms (medium and large models) by varying the depth levels of convolutional layers in the YOLOv5 algorithm using biopsied samples. We implemented data augmentation pipelines for training models, including a random translation, HSV (hue, saturation, value) color space, shear, horizontal and vertical flipping, and mosaic transformations within the window of input image size.

We detected actionable FPs in non-biopsied images using a medium baseline model and then synthesized 2.5D images via our preprocessing method for each identified lesion in the actionable images. Afterward, we fine-tuned the baseline models on an augmented image set (biopsied samples with actionable FPs added). We processed the DBT volume slice-by-slice for lesion inferencing to predict bounding boxes and corresponding scores in each slice. We then combined predictions by connecting bounding boxes along the depth via volumetric morphological closing (imclose MATLAB function, with 5 x 5 x 5 cube structure). Finally, we implemented an ensemble model by combining the fine-tuned medium and large detection models.

Data

We used the Phase 1 challenge-provided training set only, which consists of 224 total bounding boxes of the biopsy-proven lesions, including all views from 39 cancer and 62 benign studies. We subdivided our extended training set of the 2.5D images into a custom training: validation split with a ratio of 8:2 to optimize the hyperparameters for developing the detection algorithm. For training, we used YOLOv5 pretrained on the COCO dataset[46]. We used the Adam optimizer[64] to optimize the network parameters with a learning rate of 0.0005 for the baseline model and 0.00005 for fine-tuning the baseline models, along with momentum parameters of 0.937 and a weight decay of 0.0005.

B.3. Faster R-CNN Baseline Detection Model

For this paper we implemented the Faster R-CNN model[4] as a baseline model for breast lesion detection, a typical two-stage framework for object detection. Faster R-CNN is an extension of Fast R-CNN[65], which introduced a region proposal network (RPN) and applied the concept of attention in neural networks. We selected Resnet-50[35] as the feature extraction backbone, and all the hyperparameters are set as in the original work. The Phase 1 training

set was used to train the model, and we combined benign and cancer studies as one class for lesion detection. We also filtered out the lesion slices and flipped all the views onto the left side for convenience during training. We include the predictions of this model with all further results and analyses.

B.4. Technical Details

B.4.1. Detection Evaluation Metric

An algorithm's predicted lesion box was counted as a true positive detection if the pixel distance in the original image between the center of the box and the center of the lesion annotation box was less than half of (1) the diagonal length of the annotated box or (2) 100 pixels, whichever was larger. These evaluation criteria were preferred over the widely used intersection over union (IoU) metric to avoid penalizing predictions that correctly point to a lesion that may be difficult to enclose with a bounding box, or suffers from high inter-reader variability due to an uncertain boundary, spicules, or distortions. Each annotated box was assumed to span 25% of its volume's slices before and after the slice that it labels; the predicted box's slice had to have been within this range to be counted as a true positive.

For our additional analyses (Section 1.4), we used a slightly different strategy for marking lesion bounding box annotations as detected, compared to the evaluation mechanism for the challenge (Table 2) and the benchmark site (Section 1.2). Specifically, one predicted box can still detect exactly one annotated lesion box for a given algorithm. However, if the algorithm predicts a box that qualifies as detecting multiple lesions (a very rare case), the lesion box closest to the predicted box (with respect to box centroids) is marked as being detected, rather than the first of the lesion boxes according to the global list of lesion annotations; the latter was used for the challenge. Note that this distinction had little effect on results and did not change final team placements.

B.4.2. Lesion Detection Difficulty Ranking

We ranked annotated lesions in the test set by their difficulty of being detected, with the following procedure. For each annotated lesion we computed:

1. the number of algorithms that detected the lesion within score threshold corresponding to four false positive detections per DBT volume;
2. the number of false positive detections per DBT volume at score threshold corresponding to the true positive detection with the highest score, averaged over all teams that detected the lesion within four false positive detections per DBT volume.

B.4.3. Algorithm Prediction Merging Procedure

This section describes how predictions were combined from all participants, using a grid search over different methods and parameters. All possible combinations of parameters were tested in the grid search experiment, which resulted in 864 evaluations. The mean sensitivity for 1, 2, 3, and 4 FPs per volume computed on cases from all groups for each combination was then calculated as the performance metric.

Normalizing Predictions Scores Across Teams

We experimented with different score normalization methods per team to unify the score value among the teams. We attempted four methods of doing this:

1. Simple scaling, in which we divided all scores by the maximum score value to make the largest score equal to 1.
2. Scaling to a 0-1 range so that the smallest score is 0 and largest score is 1.
3. Simple percentile, in which we translated each box score to the percentile of that score among all team predictions.
4. Thresholded percentile, in which we translated each box score to the percentile of that score among team predictions higher than the score for a threshold that corresponds to 5 false positives (FPs) per volume. This method requires annotated boxes to compute transformed scores.

Our final method used the simple percentile approach (option 3).

Fusing Overlapping Bounding Boxes

The following two parameters were used for the Weighted Boxes Fusion algorithm:

© 2023 Konz N et al. *JAMA Network Open.*

1. The Intersection over Union (IoU) threshold for merging boxes. We tested values from 0.3 to 0.55 with a step size of 0.05; we used 0.4 for our final analysis.
2. The score computation method, which either allows for overflow (final score larger than 1.0) or not, if multiple boxes are predicted from the same model. More precisely, the computed score for a fused box is one of:

   1. $C = \frac{\sum_{i=1}^{T} C_i}{T} \cdot \frac{T}{N}$
   2. $C = \frac{\sum_{i=1}^{T} C_i}{T} \cdot \frac{min\ (T,N)}{N}$

   Where T is the number of fused boxes, N in the number of models, and C_i is the score from the ith model. The first equation allows for score overflow, and the second one does not; we used the second equation for our final analysis.

There is one parameter related to the three-dimensional nature of the boxes: the distance along the slice dimension between center slices to consider for two-dimensional IoU computation. We tested three possibilities: 5, 10, and 15 slices, and we finalized with 5 slices.

Finally, we adjusted the original WBF algorithm to add a voting system so that only the clusters of boxes with a minimum number of boxes are kept, and all boxes from other clusters are discarded. We tested three values for the minimum number of votes: 1, 2, and 3, and we used 1 for our final analysis.
