# Supplementary material for: A Competition, Benchmark, Code, and Data for Using Artificial Intelligence to Detect Lesions in Digital Breast Tomosynthesis
Source: JAMA Netw Open. 2023 Feb 23;6(2):e230524. doi: 10.1001/jamanetworkopen.2023.0524 (PMC9951043; doi:10.1001/jamanetworkopen.2023.0524)
Supplement: Supplement 2. — Data Sharing Statement [file jamanetwopen-e230524-s002.pdf]

Data Sharing Statement

Konz. A Competition, Benchmark, Code and Data for Using Artificial Intelligence to Detect Lesions in Digital Breast Tomosynthesis. *JAMA Netw Open*. Published February 23, 2023. doi:10.1001/jamanetworkopen.2023.0524

Data
Data available: Yes
Data types: Data (not involving human participants)
How to access data: The validation set and test set prediction data of all submitted algorithms for our challenge, for Phase 1 and Phase 2, are provided at https://wiki.cancerimagingarchive.net/pages/viewpage.action?pageId=64685580.
When available: beginning date: 03-23-2022

Supporting Documents
Document types: Statistical/analytic code
How to access documents: The code for certain submitted algorithms can be found in Table 2 of the manuscript.
When available: beginning date: 12-14-2022

Additional Information
Who can access the data: All data is publicly accessible (no application required; directly downloadable via link).
Types of analyses: Code and predictions for submitted algorithms are made available so that future researchers may base their tumor detection model development on this prior research.
Mechanisms of data availability: With investigator support.
Any additional restrictions: No restrictions; the predictions and code are completed public.
